# A Scheduling Method for Maintenance Tasks of Damaged Equipment Based on Digital Twin and Robust Optimization

**DOI:** 10.3390/s25185674

**Published:** 2025-09-11

**Authors:** Mingjie Jiang, Tiejun Jiang, Lijun Guo, Shaohua Liu

**Affiliations:** 1 Department of Management Engineering and Equipment Economics, Naval University of Engineering, Wuhan 430033, China; m23125610@nue.edu.cn (M.J.); 0907101025@nue.edu.cn (S.L.); 2 Unit No. 91976, Guangzhou 510080, China; glj726@sohu.com

**Keywords:** damaged equipment, maintenance task scheduling, digital twin, robust optimization

## Abstract

Aiming at the problems that traditional maintenance task scheduling schemes for damaged equipment have, poor adaptability to changes in uncertain factors and difficult-to-deal-with emergency scenarios, this paper proposes a maintenance task scheduling method for battle-damaged equipment based on digital twin (DT) and robust optimization. The purpose is to realize the dynamic synchronization between physical entities and virtual models through DT technology, and to leverage the anti-interference characteristics of robust optimization. The method involves constructing a multi-objective optimization model that maximizes the comprehensive importance of damaged equipment and minimizes maintenance time, and solving the model using the discrete particle swarm optimization (DPSO) algorithm. Simulation results show that this method can improve the efficiency of maintenance scheduling and the anti-interference ability in emergency situations. Through the comparison of three indicators, DT-DPSO performs the best in the maintenance scheduling of battle-damaged equipment: its convergence speed is 33.3% faster than that of DPSO and 20% faster than that of DT-non-dominated sorting genetic algorithm II (DT-NSGAII); its robustness is 16.3% higher than that of DPSO and 10.7% higher than that of DT-NSGAII; its dynamic reallocation speed is more than 40% faster than that of DPSO and more than 30% faster than that of DT-NSGAII. This method is suitable for maintenance scheduling requirements of high speed, stability, and anti-interference.

## 1. Introduction

The confrontation changes rapidly. The scheduling of damaged equipment maintenance tasks needs to closely follow the changes in the confrontation, quickly repair high-importance equipment according to real-time task requirements, and gradually restore equipment performance through batch maintenance of damaged equipment, thereby improving the overall combat capability. Faced with the problem of large demand for equipment maintenance and limited equipment maintenance support resources, it is necessary to rationally utilize maintenance resources through maintenance task scheduling to maximize the restoration of the performance of important equipment and improve the overall efficiency of equipment maintenance.

Damaged equipment maintenance task scheduling refers to the process in which the equipment supports command organization, in response to the maintenance needs of damaged equipment during task execution, conducts overall planning, dynamic allocation, and coordinated coordination of elements such as maintenance task priority, maintenance resources, maintenance processes, and time nodes, and finally realizes the efficient and orderly execution of maintenance tasks. Its goal is to complete the repair of damaged equipment in the complex confrontation in the shortest time and with the lowest resource consumption through scientific scheduling, so as to quickly restore the combat effectiveness of the equipment. At present, many scholars have studied maintenance optimization scheduling. Through comparative analysis, there are mainly the following three aspects:

(1) Optimization considering the impact of uncertain factors. The uncertainty of the maintenance system mainly comes from the fuzziness of equipment status, the randomness of fault modes, and environmental interference. Probability and statistical methods were the mainstream methods for dealing with uncertainty in the early stage. Begun et al. [1] constructed a cost-optimized probabilistic maintenance model for the uncertainty of rare failures in wind turbines, and used a Bayesian update mechanism to dynamically correct the fault probability distribution, improving decision-making robustness in small sample scenarios. Guo et al. [2] proposed a predictive Markov decision process, which handles incomplete information of multi-state systems through a partially observable Markov model (POMDP), providing a dynamic probability evaluation framework for detection and maintenance strategies. Sanchez et al. [3] optimized preventive maintenance within a limited planning horizon based on a semi-Markov process, quantifying the uncertainty of equipment degradation through a state transition probability matrix. The integrated application of intelligent algorithms has promoted the precise handling of uncertainty. Betz et al. [4] adopted a robust optimization method to deal with parameter fluctuations in facility maintenance, balancing the conservatism and economy of decisions by setting uncertainty set boundaries. In addition, fuzzy logic has become an important tool for handling subjective uncertainty. Imamguluyev [5] used a fuzzy logic model to optimize the evaluation of room maintenance factors. Xu et al. [6] effectively quantified fuzzy indicators in asphalt pavement maintenance based on a multi-attribute decision-making method with optimized triangular fuzzy numbers.
(2) Considering multi-objective optimization. Multi-objective optimization needs to balance the conflicts between objectives such as cost, reliability, and availability, and intelligent optimization algorithms have become the core tools for multi-objective decision-making. Wu et al. [7] used an improved non-dominated sorting genetic algorithm (NSGA-II) to optimize contingent maintenance of multi-unit CNC machine tool systems, while minimizing downtime and maintenance costs. Cheng et al. [8] combined NSGA-II with a Markov process to solve the multi-objective maintenance problem of airborne redundant systems operating with faults, improving system availability and reducing maintenance costs. Usman et al. [9] further integrated the improved NSGA-II algorithm with ergonomic constraints to achieve a multi-objective balance of efficiency, safety, and comfort in marine equipment maintenance scheduling. With the deepening of research, multi-attribute decision-making methods have gradually increased in complex scenarios and shown obvious advantages. Xu et al. [10] integrated three objectives of structural performance, economic cost, and environmental impact of asphalt pavement based on a multi-attribute decision-making model with optimized triangular fuzzy numbers. Khan et al. [11] designed a Fuzzy-AHP framework in information security system maintenance, converting reliability, response speed, and deployment cost into hierarchical decision indicators to improve decision consistency under multiple criteria. Guan et al. [12] integrated life cycle assessment and life cycle cost analysis to construct a dual-objective model of environment and economy for pavement preventive maintenance, providing a quantitative tool for green operation and maintenance.
(3) Considering dynamic optimization. Dynamic programming and real-time data fusion are the core technologies. Elhuseyni et al. [13] combined heuristic algorithms with dynamic programming to optimize vehicle maintenance scheduling in single dead-end track parking lots, realizing dynamic allocation of resources with time fluctuations. Van et al. [14] proposed a "predict-then-optimize" dynamic framework, which uses operational intervention data to update maintenance strategies in real time, improving the timeliness of preventive maintenance. Godoy et al. [15] introduced non-arbitrary variable intervals, and real-time corrected the evaluation of equipment status through machine learning to realize dynamic threshold adjustment of predictive maintenance. With the development of research, adaptive models and edge computing have promoted technological upgrading. Amjadian et al. [16] designed an asynchronous maintenance gap model, allowing fleet maintenance plans to be dynamically adjusted with resource availability, and handling real-time constraints through rolling horizon optimization. Szrama [17] compared the performance of autoencoders, LSTMs, and Gaussian processes in aircraft engine maintenance, and proposed a dynamically adaptive predictive maintenance model that adjusts maintenance cycles according to real-time operation data to improve the accuracy of engine remaining life prediction. Guerrero et al. [18] combined reinforcement learning with heuristic algorithms to realize real-time dynamic decision-making for maintenance of urban logistics energy systems, reducing the response time to the second level.

Through comparative analysis, the current research on maintenance optimization scheduling has the following shortcomings: First, the impact of environmental factor changes on uncertain factors has not been deeply analyzed, resulting in weak risk resistance of maintenance optimization models in specific scenarios. Second, the impact of sudden changes on uncertain factors is not considered, leading to insufficient adaptability of optimization models to dynamically changing scenarios.

**Figure 1:**
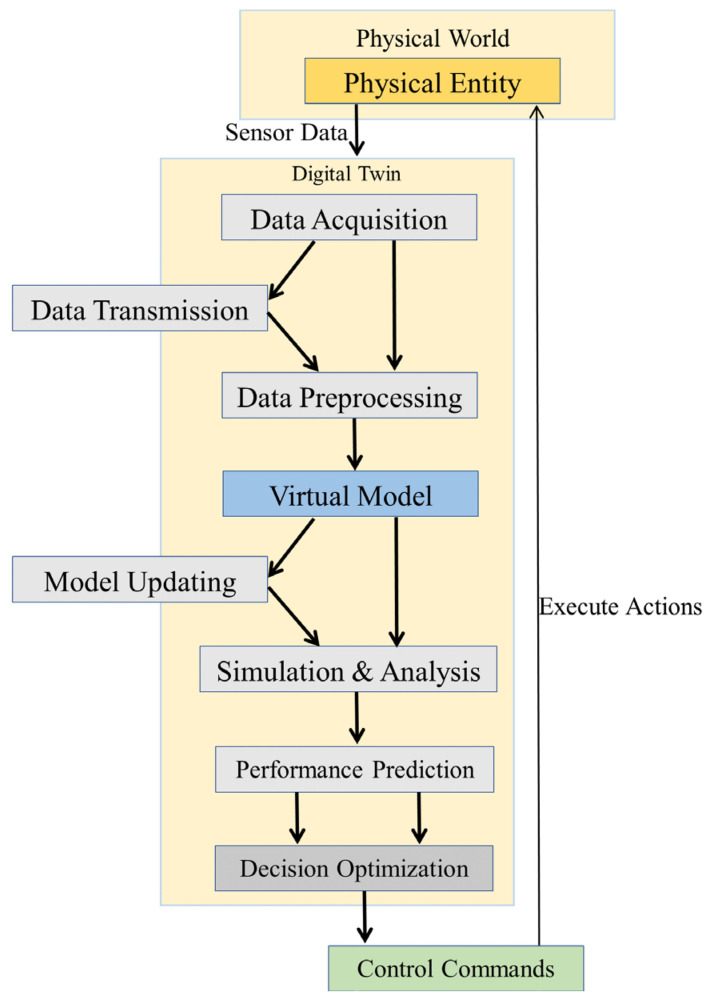
Flow chart of digital twin principle.

Digital twin [19] collects real-time status data of physical entities through sensors, the Internet of Things, and other technologies to build a data input channel for the physical world. Based on the characteristics and laws of physical entities, a virtual model is constructed to realize the mapping between physical entities and virtual models. Real-time data of physical entities is transmitted to the virtual model through communication protocols, and the analysis results of the virtual model are fed back to the physical entities, forming a closed loop of two-way data flow and dynamic state synchronization. It can simulate various scenarios of physical entities in the virtual model to realize dynamic optimization, as shown in Figure 1. Robust optimization [20,21] clarifies the optimization objectives of the system, identifies uncertain factors affecting the system, such as parameter fluctuations, external interference, model errors, interval ranges, and probability distributions, incorporates uncertain factors into the optimization model, solves the model using mathematical methods, obtains an optimization scheme for uncertain factors, and verifies the effectiveness of the robust solution through scenario testing. If the requirements are not met, model parameters are adjusted, and the optimization process is repeated to ensure that the system can still maintain basic functions in a complex environment. The combination of the two: digital twin provides scene simulation and data support for virtual–real interaction for robust optimization, and robust optimization provides anti-interference guarantee for the virtual optimization results of digital twin, jointly solving the reliable optimization problem of complex systems in dynamically uncertain environments. Digital twin and robust optimization have been widely used in combinatorial optimization problems, such as vehicle management optimization [22], medical waste delivery and transportation optimization [23], unmanned aerial vehicle trajectory optimization [24], network low-latency communication reliability optimization [25], perishable supply chain real-time optimization [26], and multi-objective optimization of urban public transport [27], showing strong risk resistance under dynamic disturbances of uncertain factors.

Aiming at the problems that traditional maintenance task scheduling schemes for damaged equipment have, poor adaptability to changes in uncertain factors and difficult-to-deal-with emergency scenarios, and combining the advantages of DT and robust optimization in solving combinatorial optimization problems, a maintenance task scheduling method for battle-damaged equipment based on DT and robust optimization is proposed. It has three main purposes: first, to realize the real-time data synchronization between physical entities and virtual models through DT technology, so as to solve the problem of information lag in traditional scheduling; second, to quantify four types of uncertain factors, including combat intensity, equipment task matching coefficient, maintenance unit fatigue coefficient and environmental threat, and construct a robust optimization model to solve the problem of poor risk resistance of traditional models; and third, to design a dynamic reallocation mechanism and a discrete particle swarm optimization algorithm for solving, so as to solve the problem of insufficient dynamic adaptability of traditional schemes. Finally, a closed loop of virtual simulation, robust solution, physical execution, and feedback optimization is formed.

## 2. Problem Description

The confrontation is complex and changeable, and the scheduling of damaged equipment maintenance tasks is greatly affected by the situation. Multiple uncertain factors continue to interfere, such as the confrontation intensity between the two sides, the matching coefficient between maintenance units and combat tasks, the fatigue coefficient of maintenance units, and the threat coefficient of the opponents deployment to maintenance units. The requirements for timeliness and accuracy of maintenance task scheduling are high, and it is impossible to adjust the scheme through multiple tests. The contradiction between the uncertainty of the confrontation and the efficiency of maintenance needs is prominent. Traditional scheduling methods are difficult to adapt to dynamically changing maintenance scenarios. The current model with known damage and fixed resources is disconnected from the dynamic confrontation, and the maintenance scheme may be "outdated as soon as it is generated". The simplified research on uncertain factors makes it difficult to adapt to changing combat scenarios. Digital twin can synchronize physical states with the virtual environment in real-time, preview scheduling schemes in virtual space to reduce trial-and-error risks, and provide dynamic data support for optimized scheduling. Robust optimization quantifies uncertainty boundaries, constructs reliable scheduling models in the worst-case scenarios, and balances efficiency and anti-interference. The combination of the two can solve the problems of information lag, weak anti-interference, and high trial-and-error risks of traditional methods, and realize efficient and reliable scheduling in complex confrontation.

Assumptions:

1. The fault types of damaged equipment are summarized into four categories: mechanical structure faults, electronic and electrical faults, energy and power faults, and task load faults. Other fault types are not studied for the time being.
2. Only the synergy importance between damaged equipment is considered, and the synergy importance between damaged equipment and non-damaged equipment is ignored.
3. A maintenance unit can only carry out maintenance on the next damaged equipment after completing the maintenance of the current one.

## 3. Modeling of Damaged Equipment Maintenance System Based on Digital Twin

A maintenance system model for damaged equipment based on DT is constructed to provide real-time data input and a foundation for virtual–real mapping for the subsequent robust optimization model.

### 3.1. Construction of Physical Model

The purpose of constructing the physical model is to establish a mapping link for entity data to provide data input for the digital twin.

1. Damaged equipment status data: $E_{i}$ is the number of the i-th damaged equipment; $D_{il}$ is the l-th fault type of the i-th damaged equipment. According to the composition structure of the equipment and common fault types, the fault types of equipment are summarized into four categories, $D_{il}=\{1,2,3,4\}$, where 1 represents mechanical structure faults, 2 represents electronic and electrical faults, 3 represents energy and power faults, and 4 represents task load faults; $r_{il}$ is the minimum skill requirement for maintaining the l-th fault type of the i-th damaged equipment; $u_{il}$ is the number of the l-th type of spare parts required for maintaining the i-th damaged equipment; $P_{i}$ is the geographical location of the i-th damaged equipment after being damaged, $P_{i}=(l_{i}^{x},l_{i}^{y})$; $N_{i}$ is the number of equipment in the team where the i-th damaged equipment is located when performing tasks; $\gamma_{i}$ is the confrontation intensity of the team where the i-th damaged equipment is located when performing tasks, $\gamma_{i}\in [0,1]$, and the value increases from low to high, indicating that the confrontation intensity gradually increases; $\theta_{i}$ is the threat coefficient when maintaining the i-th damaged equipment, $\theta_{i}\in [0,1]$.
2. Maintenance unit status data: $S_{jl}$ is the skill level of the j-th maintenance unit in maintaining the l-th fault type, $S_{jl}\in [0,1]$, which is a matrix. A larger value indicates a higher skill level and more mature technology of the maintenance unit; $P_{j}$ is the current position of the j-th maintenance unit, and $V_{j}$ is the moving speed of the j-th maintenance unit; $n_{f,j}$ is the number of damaged equipment that the j-th maintenance unit has maintained; $f_{j}$ is the fatigue coefficient of the j-th maintenance unit, which is determined by the workload completed by the maintenance unit. The more equipment maintained, the larger the fatigue coefficient of the maintenance unit; $S_{s}^{safe}$ is the safety stock of the s-th type of spare parts.
3. Environmental factor data: TR is the threat level, TR={1,2,3}, where 1 represents low threat, 2 represents medium threat, and 3 represents high threat. WH is the weather condition at the location of the damaged equipment, WH∈[0,1,2,3], corresponding to sunny, light rain, moderate rain, and heavy rain, respectively.

### 3.2. Construction of Digital Twin Model

1. Task importance evaluation model: The greater the confrontation intensity and the larger the number of equipment in the team, the more important the task performed by the damaged equipment.
  (1) $$I_{i}^{task}\;=\;\gamma_{i}\cdot N_{i},\gamma_{i}\in [0,1],N_{i}\in N^{+}$$
  where $N_{i}$ is the number of equipment in the team where the damaged equipment is located; $\gamma_{i}$ is the confrontation intensity of the team; $I_{i}^{task}$ is the task importance, that is, the importance of the task performed by the team where the damaged equipment is located in the overall task.
2. Synergy importance evaluation model: $I_{i}^{synergy}$ is the synergy importance, obtained through historical data, that is, the importance of the synergy relationship between damaged equipment i and damaged equipment k.
  (2) $$I_{i}^{synergy}=\sum_{k=1}^{m}\;C_{ik}\cdot x_{kj},c_{ik}\in [0,1]$$
3. Capability importance evaluation model:
  (3) $$I_{i}^{capacity}\;=\;\delta_{i}\cdot \eta_{i},\delta_{i}\in [0,1],\eta_{i}\in [1,10]$$
  where $\eta_{i}$ is the capability level of the damaged equipment; $\delta_{i}$ is the matching coefficient between the task performed by the damaged equipment and its own capability; $I_{i}^{capacity}$ is the capability importance of the damaged equipment.
4. Maintenance time model: The fault detection time of the damaged equipment is divided into four levels according to the four types of equipment fault types, and the sum is obtained; the spare part disassembly time and installation time are determined by the fault type and the number of required spare parts.
  (4) $$\begin{array}{c} T_{i}^{\det ection}=\sum_{l=1}^{4}t_{d,il}\;\text{..}t_{d,il}=\left\{\begin{array}{l} 2,\;\;D_{il}=1\\ 1,\;\;D_{il}=2\\ 1,\;\;D_{il}=3\\ 2,\;\;D_{il}=4 \end{array}\right.\\ T_{i}^{disassemble}=\sum_{l=1}^{4}t_{da,il}\;\text{..}t_{da,il}=\left\{\begin{array}{l} 2u_{il},\;\;D_{il}=1\\ u_{il},\;\;D_{il}=2\\ u_{il},\;\;D_{il}=3\\ 3u_{il},\;\;D_{il}=4 \end{array}\right.\\ T_{i}^{install}=\sum_{l=1}^{4}t_{r,il}\;\text{..}t_{r,il}=\left\{\begin{array}{l} 2u_{il},\;\;D_{il}=1\\ u_{il},\;\;D_{il}=2\\ u_{il},D_{il}=3\\ u_{il},\;\;D_{il}=4 \end{array}\right. \end{array}$$
  where $T_{i}^{\det ection}$ is the fault detection time; $T_{i}^{disassemble}$ is spare part disassembly time; $T_{i}^{install}$ is spare part installation time; $t_{d,il}$ is the detection time required for the l-th fault of the i-th damaged equipment; $t_{da,il}$ is the single spare part disassembly time required for the l-th fault of the i-th damaged equipment; $t_{r,il}$ is the single spare part installation time required for the l-th fault of the i-th damaged equipment.
5. Maneuvering time model: The maneuvering time consists of three parts: the maintenance unit maneuvering to the fault point of the damaged equipment, the sequential maintenance of the fault points of the damaged equipment, and the last fault point of the damaged equipment to the starting point of the maintenance unit.
  (5) $$\begin{array}{l} T_{ij}^{move}=(\sqrt{{\left(l_{j}^{x}-l_{i}^{x}\right)}^{2}+{\left(l_{j}^{y}-l_{i}^{y}\right)}^{2}}\\ +\sum_{i=1}^{m}\sqrt{{\left(l_{i}^{x}-l_{i+1}^{x}\right)}^{2}+{\left(l_{i}^{y}-l_{i+1}^{y}\right)}^{2}}\\ +\sqrt{{\left(l_{m}^{x}-l_{j}^{x}\right)}^{2}+{\left(l_{m}^{y}-l_{j}^{y}\right)}^{2}})/v_{j}\\ l_{i}^{x},\;\;l_{j}^{x},\;\;l_{i}^{y},\;\;l_{j}^{y}\in (0,40) \end{array}$$
  where $P_{j}=(l_{j}^{x},l_{j}^{y})$ is the position information of the maintenance unit, $P_{i}=(l_{i}^{x},l_{i}^{y})$ is the position information of the damaged equipment; $v_{j}$ is the maneuvering speed of the maintenance unit. The unit of position coordinates is kilometers, and the unit of speed is minutes per kilometer.
6. Fatigue impact coefficient model:
  (6) $$f_{j}=\left\{\begin{array}{l} 0.1,n_{f,j}=1\\ 0.2,n_{f,j}=2\\ 0.35,n_{f,j}=3\\ 0.5,4\leq n_{f,j}\leq 5 \end{array}\right.$$
  where $n_{f,j}$ is the number of damaged equipment that the maintenance unit has maintained, and $f_{j}$ is the fatigue impact coefficient.
7. Threat coefficient model:
  (7) $$\theta_{i}=\left\{\begin{array}{l} 0.1,\;\;TR=1\\ 0.2,\;\;TR=2\\ 0.4,\;\;TR=3 \end{array}\right.$$
  where TR is the threat level at the location of the damaged equipment and $\theta_{i}$ is the threat coefficient.
8. Weather impact coefficient model:
  (8) $$h_{i}^{wh}=\left\{\begin{array}{l} 0,\;\;WH=0\\ 1/3,\;\;WH=1\\ 2/3,\;\;WH=2\\ 4/5,\;\;WH=3 \end{array}\right.{\bar{f}}_{j,new}^{\max}={\bar{f}}_{j}^{\max}\cdot (1-h_{i}^{wh})$$
  where WH is the weather condition at the location of the damaged equipment; $h_{i}^{wh}$ is the weather impact coefficient. ${\bar{f}}_{j}^{\max}$ is the maximum fatigue coefficient that the maintenance unit can bear.

In the scenario of maintenance scheduling for battle-damaged equipment during wartime, digital twins need to achieve highly reliable synchronization between physical entities and virtual models under complex electromagnetic environments and dynamic battlefield situations. The core objectives are to rapidly locate battle-damaged equipment, accurately assess damage levels, dynamically match spare part resources, and support decision-making for maintenance task scheduling. Combined with the characteristics of wartime environments, the quantitative verification results of synchronization accuracy and sensor technical parameters are presented as follows:

### 3.3. Synchronization Accuracy and Sensor Technical Parameters

Quantitative Verification Results of Synchronization Accuracy

1. Error Rate

1. Error rate of position information synchronization: Real-time positioning of battle-damaged equipment needs to resist electromagnetic interference, adopting Beidou and Inertial Navigation System (INS) combined positioning. Without strong electromagnetic interference, the positioning error rate is ≤0.5%; under strong electromagnetic interference, the positioning error rate with INS assistance is ≤3%.
2. Error rate of damage level assessment: For typical battle damages such as engine abnormal noise and circuit short circuits, verification is conducted through multi-sensor fusion.
3. Functional damage level: The error rate of joint determination by vibration and current sensors is ≤1.5%; for internal component damage, the error rate of matching between ultrasonic flaw detection and virtual models is ≤3%, and internal cracks larger than 0.5 mm can be identified.
4. Error rate of spare part demand identification: Through the synergy of visual recognition and RFID tags, the matching between spare part models and battle-damaged parts is realized. The error rate of spare part model identification is ≤0.8%; the synchronization error rate between spare part inventory and virtual models is ≤1%, ensuring that the spare parts carried by maintenance units fully match the demands.

2. Time Delay

1. Data transmission delay: Considering tactical-level anti-jamming communication, the transmission delay of battle-damage data for a single piece of equipment is ≤200 ms; during concurrent transmission of multiple pieces of equipment, the aggregation delay at edge nodes is ≤500 ms to avoid data congestion affecting scheduling.
2. Virtual model update delay: During dynamic changes in battle-damage status, the dynamic update delay of position coordinates is ≤100 ms, ensuring real-time alignment between the virtual twin and the motion trajectory of the physical equipment; the iterative update delay of damage levels is ≤300 ms.
3. Maintenance scheduling decision delay: The full-link delay from battle-damage data collection to maintenance task allocation: the delay in generating maintenance plans for a single piece of equipment is ≤5 s, including spare part matching and path planning for maintenance units; the delay in priority ranking and resource allocation for multiple pieces of equipment is ≤10 s, supporting parallel scheduling of 10–20 battle-damaged pieces of equipment.

3. Sensor Types and Sampling Frequencies in Wartime Scenarios

1. Beidou-3 + INS combined navigation module: Used for real-time positioning of battle-damaged equipment, resistant to 1500 V electromagnetic pulses, with an operating temperature range of −40 °C~70 °C, and a sampling frequency of 10 Hz in dynamic state and 1 Hz in static state.
2. Anti-jamming vibration acceleration sensor: Used for monitoring internal damage of equipment, with a measurement range of ±100 g, oil and dust proof, supporting active noise reduction, and a sampling frequency of 5 kHz.
3. Industrial-grade RFID reader–writer: Used for spare part model identification and inventory synchronization, capable of penetrating metal, resistant to electromagnetic shielding, with a sampling frequency of 10 Hz.

## 4. Multi-Objective Robust Optimization Model

A multi-objective robust optimization model is constructed, with clear boundaries of uncertain factors and optimization objectives, to provide support for scheduling schemes.

### 4.1. Construction of Uncertainty Set

Considering that in the confrontation process, the confrontation intensity between both sides, the task matching coefficient of equipment, the fatigue coefficient of maintenance units, and the threat coefficient of the environment have certain fluctuation ranges, an uncertainty set is constructed based on the fluctuation ranges of these four uncertain factors. 

To ensure robustness during objective optimization: the confrontation intensity and equipment task matching degree, which are related to equipment importance, are calculated using the minimum values within their fluctuation ranges; the threat coefficient, which is related to maintenance time, is calculated using the maximum value within its fluctuation range; and the fatigue coefficient is calculated according to the actual number of equipment under maintenance.

(9) $$\Gamma =\left\{\tilde{\gamma_{i}}\in [\gamma_{i}^{\min},\gamma_{i}^{\max}],\;\tilde{\delta_{i}}\in [\delta_{i}^{\min},\delta_{i}^{\max}],\;\;\tilde{f_{j}}\in [f_{j}^{\min},f_{j}^{\max}],\;\tilde{\theta_{i}}\in [\theta_{i}^{\min},\theta_{i}^{\max}]\right\}$$

In the formula: $\tilde{\gamma_{i}}$ is the fluctuation range of the confrontation intensity of the i-th damaged equipment, $\gamma_{i}^{\min}$ is the minimum confrontation intensity, and $\gamma_{i}^{\max}$ is the maximum confrontation intensity; $\tilde{\delta_{i}}$ is the fluctuation range of the task matching degree of the i-th damaged equipment, $\delta_{i}^{\min}$ is the minimum task matching degree, and $\delta_{i}^{\max}$ is the maximum task matching degree; $\tilde{f_{j}}$ is the fluctuation range of the fatigue coefficient of the j-th maintenance unit; $f_{j}^{\min}$ is the minimum fatigue coefficient; $f_{j}^{\max}$ is the maximum fatigue coefficient; $\tilde{\theta_{i}}$ is the fluctuation range of the threat coefficient when maintaining the i-th damaged equipment; $\theta_{i}^{\min}$ is the minimum threat coefficient; $\theta_{i}^{\max}$ is the maximum threat coefficient.

### 4.2. Robust Objective Functions

Objective 1: Maximize the comprehensive importance of damaged equipment.

When scheduling damaged equipment maintenance tasks, priority is given to damaged equipment that contributes greatly to the current situation and meets the operational capability requirements. Maximizing the comprehensive importance of damaged equipment means maximizing the sum of the comprehensive importance of the maintained damaged equipment. The comprehensive importance of damaged equipment consists of three parts: the importance of the task performed by the damaged equipment, the importance of the synergy between damaged equipment, and the capability importance of the damaged equipment.

(10) $$\begin{array}{c} Max\;Z_{1}=\sum_{i=1}^{m}I_{i}^{cph}x_{ij}\\ 1\leq i\leq m,1\leq j\leq n\\ I_{i}^{cph}=\phi_{1}I_{i}^{task}+\phi_{2}I_{i}^{synergy}+\phi_{3}I_{i}^{capacity}\\ I_{i}^{cph},\;\;I_{i}^{task},\;\;I_{i}^{synergy},\;\;I_{i}^{capacity}\in [0,1]\\ \phi_{1}+\phi_{2}+\phi_{3}=1 \end{array}$$

where $x_{ij}$ is the decision variable, indicating whether the j-th maintenance unit maintains the i-th damaged equipment, where 1 means maintenance and 0 means no maintenance; $I_{i}^{cph}$ is the comprehensive importance of the i-th damaged equipment; $I_{i}^{task}$ is the importance of the task performed by the team where the i-th damaged equipment is located in the overall task; $I_{i}^{synergy}$ is the synergy importance of the i-th damaged equipment; $I_{i}^{capacity}$ is the capability importance of the i-th damaged equipment; $\phi_{1},\;\phi_{2},\;\phi_{3}$ are the weights of importance. Considering that the equipment’s own capability is more important, the weight of the equipment’s capability importance is larger, so the weights are 0.3, 0.3, and 0.4, respectively.

Objective 2: Minimize the maximum maintenance time of maintenance units.

In confrontation, maintenance time should be shortened as much as possible to enable important equipment to be put into use quickly. Minimizing the maximum maintenance time of maintenance units means minimizing the maximum time among multiple parallel maintenance units. The total maintenance time consists of comprehensive maintenance time, fatigue impact time of maintenance units, and threat impact time; the comprehensive maintenance time of damaged equipment consists of fault detection time, spare part disassembly time, spare part installation time, and maneuvering time; the fatigue impact time of maintenance units is determined by the maintenance tasks completed by the maintenance units; the threat impact time is determined by the threat level at the location of the equipment.

(11) $$\begin{array}{l} Min\;Z_{2}=\max_{j=1}^{n}\left(\sum_{i=1}^{m}T_{ij}^{cph}(1+f_{j}+\theta_{i})\cdot x_{ij}\right)\\ 1\leq i\leq m,1\leq j\leq n\\ T_{ij}^{cph}=T_{i}^{\det ection}+T_{i}^{disassembly}+T_{i}^{install}+T_{ij}^{move} \end{array}$$

where $T_{ij}^{cph}$ is the comprehensive time used by the j-th maintenance unit to maintain the i-th damaged equipment; $T_{i}^{\det ection}$ is the detection time required for the i-th damaged equipment; $T_{i}^{disassemble}$ is the spare part disassembly time required for the i-th damaged equipment; $T_{i}^{install}$ is the spare part installation time required for the i-th damaged equipment; $T_{ij}^{move}$ is the total maneuvering time required for the j-th maintenance unit to maintain the damaged equipment it is responsible for, and the time is in minutes.

### 4.3. Robust Constraints

1. The maintenance skill of the maintenance unit should be greater than the minimum skill requirement of the damaged equipment.
  (12) $$r_{il}\cdot x_{ij}\leq S_{jl},\;\forall i,j,l,\;\;r_{il}\in [0,1]$$
  where $S_{jl}$ is the skill level of the j-th maintenance unit in maintaining the l-th fault type, and $r_{il}$ is the minimum skill requirement for maintaining the l-th fault type of the i-th damaged equipment.
2. Each damaged equipment can only be assigned to one maintenance unit for maintenance.
  (13) $$\sum_{j=1}^{n}x_{ij}=1,\;\forall i$$
3. The total number of spare parts required by all damaged equipment cannot exceed the spare part inventory.
  (14) $$\sum_{i=1}^{m}\sum_{l=1}^{4}u_{il}x_{ij}\leq S_{s}^{safe},\;\forall j,s$$
  where $u_{il}$ is the number of the s-th type of spare parts required for maintaining the i-th damaged equipment; $S_{s}^{safe}$ is the safety stock of the s-th type of spare parts.
4. The threat to all maintenance units during maintenance cannot exceed the maximum threat coefficient of the equipment they are responsible for. The threat coefficient takes the maximum value in the interval to ensure that the upper limit of the threat borne by the maintenance unit does not exceed the threat threshold.
  (15) $$\max(\theta_{i}^{\max}x_{ij})\leq {\bar{\theta_{j}}}^{\max},\;\forall j\;{\bar{\theta }}_{j}\in [0,1]$$
  where ${\bar{\theta }}_{j}^{\max}$ is the threat threshold that the j-th maintenance unit can bear.
5. The fatigue degree of all maintenance units does not exceed the fatigue threshold. The fatigue coefficient takes the maximum value in the interval to ensure that the upper limit of the fatigue borne by the maintenance unit does not exceed the threshold.
  (16) $$f_{j}^{\max}\leq {\bar{f_{j}}}^{\max},\;\forall j\;\bar{f_{j}}\in [0,1]$$
  where ${\bar{f_{j}}}^{\max}$ is the fatigue coefficient threshold that the j-th maintenance unit can bear.

## 5. Solution with Discrete Particle Swarm Optimization Algorithm

Discrete particle swarm optimization (DPSO) is an extension of the particle swarm optimization algorithm in discrete space. Its core is to convert continuous positions and velocities into discrete forms to adapt to discrete variables. Compared with traditional discrete optimization algorithms, it can better balance local search and global search and efficiently solve the scheduling problem of damaged equipment maintenance tasks.

1. Particle Coding and Initialization Optimization

An integer coding method of “maintenance unit-damaged equipment” mapping is adopted. The particle dimension is the total number of damaged equipment, and the value range of each dimension is the set of maintenance unit numbers, {1,2,…,n}. For example, the particle [3, 1, 2, 3] indicates that the first damaged equipment is responsible for by maintenance unit 3, the second by maintenance unit 1, the third by maintenance unit 2, and the fourth by maintenance unit 3. The coding must satisfy the ‘one-to-one’ constraint, that is, each damaged equipment is only assigned to one maintenance unit, and the initial assignment needs to check the skill matching of the maintenance unit in advance, that is, the skill level of the maintenance unit meets the minimum skill requirement of the damaged equipment.

Constraint-oriented initialization: To avoid frequent violation of constraints such as skill mismatch and insufficient spare parts when randomly generating particles, a two-step initialization method is adopted as follows: the first is the pre-assignment stage, in which maintenance units that meet the skill requirements are screened as candidates according to the fault type of the damaged equipment; the second is the random sampling stage, in which each damaged equipment is randomly assigned a maintenance unit from the candidate set, and the spare part inventory constraint is checked in real time. If violated, sampling from the candidate set is re-conducted until an initial particle swarm that meets all constraints is generated.

2. Objective Function and Fitness Calculation

When converting the weighted sum of dual objectives into a single-objective fitness function, the setting of weight coefficients needs to be dynamically adjusted in combination with battlefield priorities: When the battlefield confrontation intensity is high, such as $\gamma_{i}\geq 0.7$, it is necessary to prioritize the maintenance of core equipment and ensure the maximization of comprehensive importance, so the weights are set as $\omega_{1}=0.6$ and $\omega_{2}=0.4$; when the confrontation intensity is low, such as $\gamma_{i}<0.5$, it is necessary to quickly restore equipment capabilities for rapid deployment, focusing on minimizing maintenance time, so the weights are set as $\omega_{1}=0.4$ and $\omega_{2}=0.6$. In this paper, for the conventional scenario, importance and maintenance time are equally important, with weights set as $\omega_{1}=0.5$ and $\omega_{2}=0.5$.

The fitness calculation formula is modified, and dimension differences are eliminated through normalization to ensure that the double objectives are balanced in fitness.

(17) $$Fitness=\omega_{1}\cdot \frac{\sum x_{ij}\cdot I_{i}}{\max(I_{i})}\omega_{2}\cdot \frac{\max(\sum x_{ij}\cdot T_{ij})}{\max(T_{ij})}$$

where $\max(I_{i})$ is the maximum value of the comprehensive importance of all damaged equipment, and $\max(T_{ij})$ is the maximum possible maintenance time of a single maintenance unit.

3. Iterative Optimization

Speed and position update strategies:

The speed update adopts an integer PSO model, and the formula is

(18) $$v_{k+1}=\omega_{p}\left(v_{k}+c_{1}r_{1}(pbest_{k}x_{k})+c_{2}r_{2}(gbestx_{k})\right)$$

where $\omega_{p}$ is the inertia weight, balancing global and local searches; $c_{1},\;c_{2}$ are learning factors; $r_{1},r_{2}\in (0,1)$ are random numbers. The speed value represents the adjustment tendency of maintenance unit assignment, with positive values indicating approaching excellent solutions and negative values indicating exploring new assignments. 

The position update maps the speed to the adjustment probability through the sigmoid function:

(19) $$x_{k+1}=\left\{\begin{array}{ll} n_{new} & \mathrm{if}\;\mathrm{rand}()<\frac{1}{1+e^{-v_{k+1}}}\\ x_{k} & \mathrm{otherwise} \end{array}\right.$$

where $n_{new}$ is the new maintenance unit number. It is randomly selected from the candidate set to ensure that the updated one still meets the skill and spare part constraints.

Constraint repair mechanism: If the updated position violates the constraints, a repair process is initiated to retain the assignments that meet the constraints. For damaged equipment that violates the constraints, based on the pbest record, the best-performing maintenance unit in the latest iteration is re-assigned from the candidate set to reduce invalid searches.

Adaptive parameter adjustment: When the gbest is not updated for five consecutive generations, a disturbance operation is triggered, randomly selecting 20% of the particles and randomly resetting 30% of their dimensions to avoid falling into local optima.

4. Iteration Termination and Result Verification

Termination conditions: The maximum number of iterations is set to 200, or the iteration is terminated early when the fitness value of the global optimal solution (gbest) fluctuates by less than 0.01 for 10 consecutive generations, balancing solution accuracy and time cost.

Result verification: After outputting the assignment scheme corresponding to gbest, secondary verification of all constraints is required. If there are slight violations, adjustments are made through digital twin virtual scene simulation to ensure the feasibility of the scheme.

**Figure 2:**
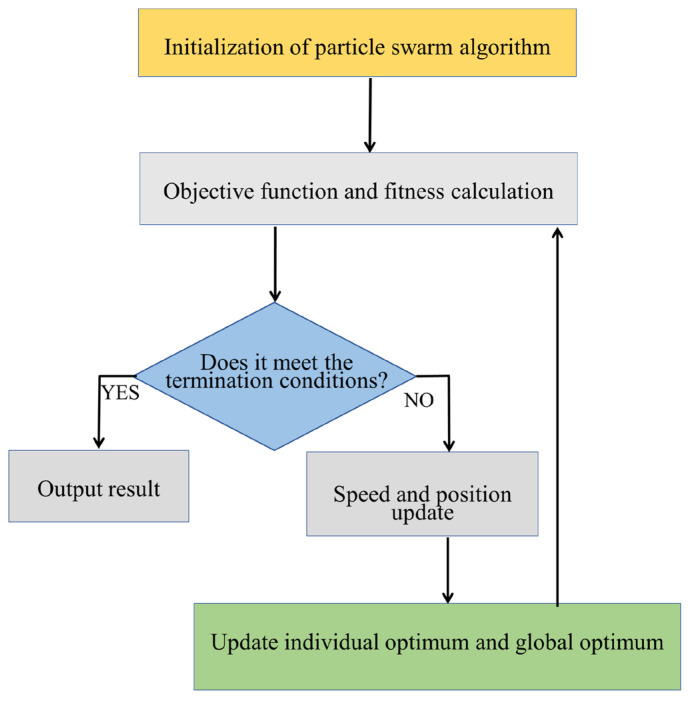
Flowchart of implementation process of discrete particle swarm optimization algorithm.

The discrete particle swarm optimization algorithm is used to solve the model, and the implementation process is shown in Figure 2.

## 6. Dynamic Reallocation Implementation Process

When the opponent adjusts its deployment, the threat coefficient will change; when there is windy and rainy weather, the maintenance unit is affected by the weather, and the fatigue coefficient threshold will decrease; when new damaged equipment is added, the equipment importance and maintenance time will change. Therefore, re-optimization scheduling is initiated when one of the three conditions is met: the opponent adjusts its deployment, there is windy and rainy weather, or new damaged equipment is added. The specific process is as follows:

1. Real-time data collection and update

Relying on the IoT perception layer of the digital twin, key data are collected as follows:

Damaged equipment: fault types, positions, and comprehensive importance of new equipment; remaining maintenance time of existing damaged equipment. Maintenance units: real-time positions, current fatigue coefficients, number of maintained equipment, and remaining spare parts. Environment: weather conditions and threat conditions.

2. Key steps of model reconstruction

Recalculation of comprehensive importance: For newly added damaged equipment, calculate its task importance, synergy importance, and capability importance; for existing damaged equipment, if the synergy relationship is affected by the newly added equipment, recalculate the synergy importance. Recalculation of maintenance time: when affected by weather, the fatigue coefficient is recalculated; when affected by threats, the threat coefficient is recalculated.

3. Rapid iteration of robust re-optimization

First, update of the uncertainty set: adjust the fluctuation ranges of the four uncertain factors based on new data, and reduce noise impact using a sliding window mechanism. Second, adaptation of the re-optimization algorithm: to shorten the response time, the re-optimization reuses the pbest and gbest historical records of the initial optimization, reduces the population size to 50, and reduces the maximum number of iterations to 100, compressing the solution time to 60% of the initial optimization through this method. Third, priority ranking of schemes: output three candidate schemes based on fitness ranking, simulate the execution effect of each scheme in 10 virtual scenarios through the digital twin, and select the scheme with the highest robustness score as the final decision.

**Figure 3:**
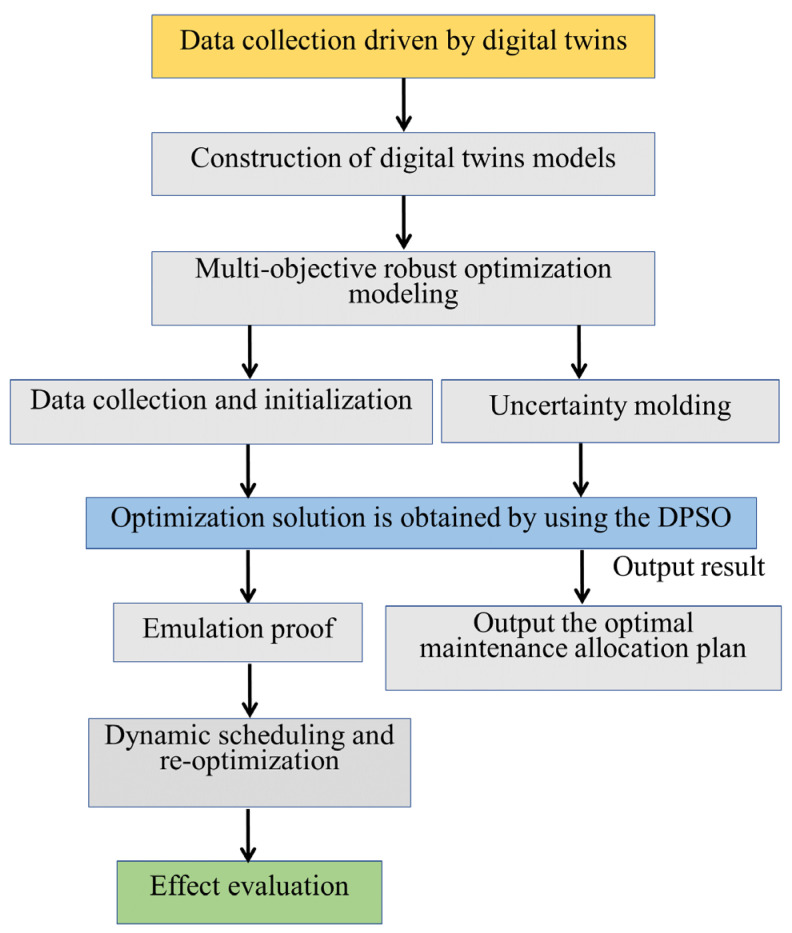
Overall implementation process.

The overall implementation process of damaged equipment maintenance task scheduling is shown in Figure 3.

## 7. Simulation Verification

The simulated physical model is built in the equipment maintenance simulation system, with 12 damaged equipment and 4 newly added damaged equipment later. Three maintenance units are arranged for emergency repair. The relevant parameters were obtained from Formulas (1)–(8), as shown in Table 1, Table 2, Table 3 and Table 4. The maintenance task scheme needs to be dynamically adjusted using digital twin and robust optimization methods according to real-time conditions.

Data sources: The status data of damaged equipment are obtained from the historical equipment maintenance database. Among them, the range of combat intensity and the range of threat coefficient are set based on the statistical data of combat scenarios. The data of maintenance units are derived from the skill assessment results of maintenance personnel. Among them, the mobile speed refers to the actual mobile capability test data of maintenance units; the maximum fatigue coefficient is obtained from the results of continuous operation fatigue experiments of maintenance personnel. The weather conditions and threat levels in environmental data refer to the evaluation standards of environmental impact in combat environments.

The maneuvering speed of the three maintenance units is set to 1 km/min, the threat safety threshold that the maintenance units can bear is 0.6, and the maximum fatigue coefficient that the maintenance units can bear is 0.6. From Formula (9), the overall fluctuation range of the threat coefficient at the location of the damaged equipment is [0.1, 0.5], and the fluctuation range of the threat coefficient at each point is within the overall fluctuation range; the overall fluctuation range of confrontation intensity is [0.2, 1], and the confrontation intensity range of each damaged equipment is within the overall fluctuation range; the overall fluctuation range of the task matching coefficient is [0.3, 1], and the task matching coefficient range of each damaged equipment is within the overall fluctuation range; the fluctuation range of the fatigue coefficient of the maintenance unit is [0.1, 0.5], which is determined according to the actual number of maintained equipment during calculation. The mark (√) after the equipment indicates that the damaged equipment has been maintained in the previous scheme.

### 7.1. Initial Data

1. Parameters of damaged equipment
  **Table 1:** Parameters of damaged equipment.

Number	Fault Type	Skill Requirement	Spare Part Demand	Position	Number of Equipment in Team	Confrontation Intensity	Capability Level	Task Matching Coefficient	Threat Coefficient
E1	2, 4	0.4, 0.4	2, 3	(10, 20)	5	[0.5, 0.6]	3	[0.5, 0.7]	[0.1, 0.3]
E2	1	0.6	4	(15, 20)	8	[0.6, 0.8]	8	[0.3, 0.4]	[0.1, 0.15]
E3	1, 3	0.3, 0.5	2, 3	(20, 25)	3	[0.3, 0.5]	2	[0.7, 0.9]	[0.1, 0.2]
E4	1	0.5	4	(5, 15)	6	[0.8, 0.9]	7	[0.9, 0.95]	[0.1, 0.15]
E5	1, 2	0.2, 0.3	2, 4	(10, 25)	4	[0.4, 0.6]	4	[0.4, 0.5]	[0.2, 0.5]
E6	2	0.3	4	(10, 15)	7	[0.7, 0.8]	6	[0.9, 0.95]	[0.1, 0.2]
E7	2	0.4	3	(20, 30)	5	[0.2, 0.4]	1	[0.6, 0.8]	[0.1, 0.15]
E8	4	0.6	3	(5, 5)	5	[0.4, 0.6]	4	[0.7, 0.8]	[0.1, 0.5]
E9	2, 3	0.2, 0.4	2, 4	(5, 10)	9	[0.9, 0.95]	5	[0.5, 0.6]	[0.3, 0.4]
E10	2	0.4	3	(15, 15)	6	[0.4, 0.5]	3	[0.8, 0.9]	[0.1, 0.2]
E11	1, 2	0.2, 0.6	2, 2	(8, 22)	3	[0.3, 0.5]	4	[0.3, 0.4]	[0.3, 0.5]
E12	4	0.7	2	(8, 10)	3	[0.3, 0.4]	3	[0.2, 0.3]	[0.3, 0.4]

2. Parameters of maintenance units
  **Table 2:** Parameters of maintenance units.

Number	Skill Level Matrix (Corresponding to Faults 1–4)	Position	Speed (km/min)	Maximum Fatigue Coefficient	Spare Part Safety Stock (Corresponding to Fault Types 1–4)
A	[0.5, 0.6, 0.3, 0.4]	(10, 18)	0.5	0.5	[4, 7, 2, 3]
B	[0.5, 0.6, 0.5, 0.6]	(18, 10)	0.6	0.5	[3, 6, 8, 3]
C	[0.5, 0.6, 0.5, 0.6]	(20, 15)	0.4	0.5	[3, 8, 4, 3]

3. Parameters of newly added damaged equipment
  **Table 3:** Parameters of newly added damaged equipment.

Number	Fault Type	Skill Requirement	Spare Part Demand	Position	Number of Equipment in Team	Confrontation Intensity	Capability Level	Task Matching Coefficient	Threat Coefficient
E13	3, 4	0.5, 0.4	3, 3	(8, 22)	4	[0.3, 0.4]	4	[0.4, 0.5]	[0.1, 0.3]
E14	4	0.4	3	(12, 8)	5	[0.5, 0.6]	6	[0.2, 0.4]	[0.1, 0.15]
E15	1, 3	0.3, 0.5	1, 3	(17, 19)	4	[0.3, 0.5]	4	[0.6, 0.7]	[0.1, 0.2]
E16	2	0.7	4	(16, 23)	4	[0.3, 0.5]	4	[0.4, 0.5]	[0.3, 0.5]

4. Synergy importance matrix of damaged equipment
  **Table 4:** Synergy importance data of damaged equipment.

	E1	E2	E3	E4	E5	E6	E7	E8	E9	E10	E11	E12	E13	E14	E15	E16
E1	0	0.4	0.2	0	0.7	0	0.1	0	0	0	0	0	0	0	0	0
E2	0.4	0	0.3	0	0.3	0	0	0	0	0.8	0	0	0	0	0.3	0
E3	0.2	0.3	0	0	0	0	0.6	0	0	0	0	0	0	0	0	0
E4	0	0	0	0	0.5	0	0	0	0.8	0	0	0	0	0.6	0	0
E5	0.7	0.3	0	0	0	0	0.3	0	0	0	0	0	0	0	0	0
E6	0	0	0	0.5	0	0	0	0.4	0.4	0	0	0	0.4	0	0	0
E7	0.1	0	0.6	0	0.3	0	0	0	0	0	0	0	0	0	0	0
E8	0	0	0	0	0	0.4	0	0	0.7	0	0	0	0	0	0	0
E9	0	0	0	0.8	0	0.4	0	0.7	0	0	0	0	0.3	0	0	0
E10	0	0.8	0	0	0	0	0	0	0	0	0	0	0	0	0	0
E11	0	0	0	0	0	0	0	0	0	0	0	0.3	0	0	0	0
E12	0	0	0	0	0	0	0	0	0	0	0.3	0	0	0	0	0
E13	0	0	0	0	0	0.4	0	0	0.3	0	0	0	0	0.2	0	0.3
E14	0	0	0	0.6	0	0	0	0	0	0	0	0	0.2	0	0.2	0
E15	0	0.3	0	0	0	0	0	0	0	0	0	0	0	0.2	0	0
E16	0	0	0	0	0	0	0	0	0	0	0	0	0.3	0	0	0

### 7.2. Initial Allocation Scheme

According to the maintenance task objectives, priority is given to damaged equipment with high comprehensive importance and less maintenance time, and all maintenance tasks are ensured to meet relevant constraints., the allocation results are derived from Formulas (10)–(19), as shown in Table 5.

### 7.3. Dynamic Reallocation Schemes Under Three Scenarios

Scenario 1: The opponent adjusts deployment.

After each of the three maintenance units has maintained the first equipment, the opponent adjusts its deployment and increases the attack intensity on the area where the maintenance units perform tasks. The threat thresholds that the three maintenance units can bear drop to 0.2, 0.3, and 0.4, respectively. The allocation results are shown in Table 6.

Scenario 2: Occurrence of windy and rainy weather.

After each of the three maintenance units has maintained the second equipment, the threat thresholds that the three maintenance units can bear return to the initial state. At this time, windy and rainy weather occurs, which has a great impact on the maintenance capability of the maintenance units. The locations of maintenance units A and B are in light rain, and the location of maintenance unit C is in moderate rain. Due to the good protective measures of maintenance unit A, its maximum bearable fatigue coefficient remains unchanged, while the maximum bearable fatigue coefficients of maintenance units B and C drop to 0.4 and 0.2, respectively. The allocation results are shown in Table 7.

Scenario 3: Addition of new damaged equipment.

After each of the three maintenance units has maintained the third equipment, four important damaged equipment are suddenly added, numbered E13-E16. At this time, the windy and rainy weather stops, and the maximum bearable fatigue coefficients of the three maintenance units return to the initial level. The allocation results are shown in Table 8.

**Figure 4:**
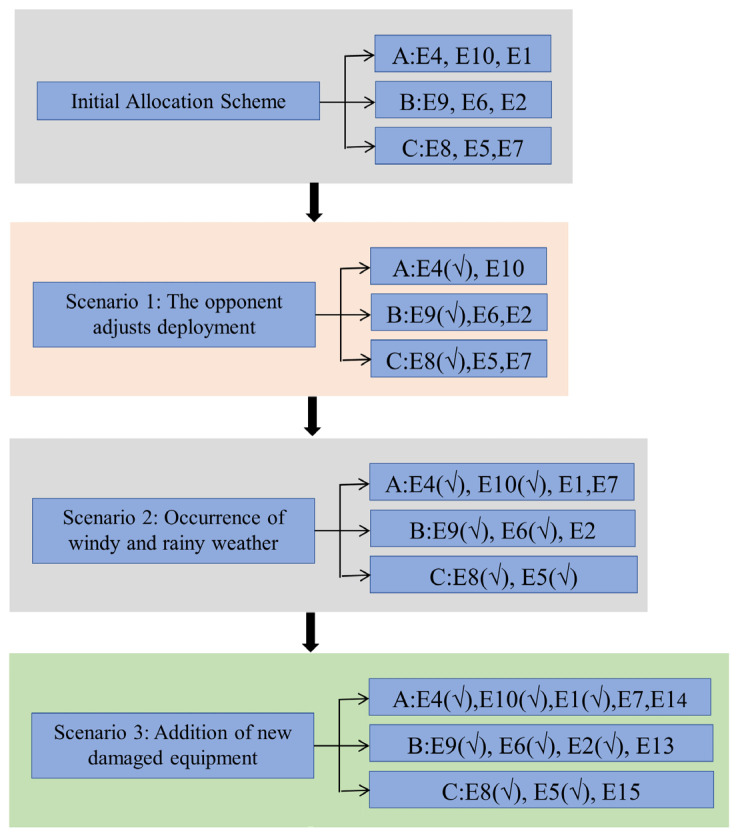
Flowchart for dynamic reallocation in different scenarios.

The primary distribution and distribution in different scenarios are shown in Figure 4.

### 7.4. Algorithm Performance Testing

To verify the superiority of the proposed digital twin–discrete particle swarm optimization (DT-DPSO) algorithm in wartime wartorn equipment maintenance task scheduling, two mainstream comparative algorithms were selected, and tests were conducted from three indicators: convergence speed, robustness, and dynamic reallocation speed. All algorithms were run under the same hardware environment (Intel Core i7-12700H, 16GB DDR4, Windows 10 system) and software platform (MATLAB R2023a) to ensure the fairness of the comparison.

1. Basis for Selecting Comparative Algorithms

Combined with the characteristics of wartime maintenance scheduling scenarios and relevant research results, two comparative algorithms were selected as follows:

Traditional discrete particle swarm optimization (DPSO): It does not integrate digital twin technology and only adopts basic discrete particle swarm optimization. It is used to verify the improvement effect of digital twin real-time data support on algorithm performance and is a classic algorithm for discrete space scheduling problems.

Digital twin–non-dominated sorting genetic algorithm (DT-NSGAⅡ): A multi-objective optimization algorithm integrated with digital twin technology. Referring to the application of NSGA-II in multi-objective scheduling of equipment maintenance in Reference 8, it is used to compare the adaptability differences between the particle swarm search mechanism and the genetic algorithm evolution mechanism in this scenario.

2. Algorithm Parameter Settings

To eliminate the interference of parameter differences on the test results, the main parameters of the three algorithms were kept consistent, and only algorithm-specific parameters were set differently. The details are shown in Table 9:

3. Multi-Indicator Performance Comparison Tests

(1) Convergence Speed Comparison

Convergence speed reflects the ability of an algorithm to quickly find the optimal scheduling scheme. It is evaluated by three sub-indicators: the number of iterations to reach stable fitness, convergence accuracy, and initial allocation calculation time. The results are shown in Table 10:

(2) Robustness Comparison

Robustness reflects the stability of the algorithms scheme under battlefield uncertainties factors. It is evaluated by the standard deviation of total equipment importance, the standard deviation of maximum maintenance time, and the scheme feasibility rate under uncertain disturbances. The results are shown in Table 11:

(3) Dynamic Reallocation Speed Comparison

Dynamic reallocation speed reflects the algorithms response ability to battlefield mutations (new wartorn equipment, enemy deployment adjustments, windy and rainy weather). The reallocation calculation times under three typical mutation scenarios were tested, and the results are shown in Table 12:

## 8. Result Analysis

1. Analysis of total equipment importance and maximum maintenance time

**Figure 5:**
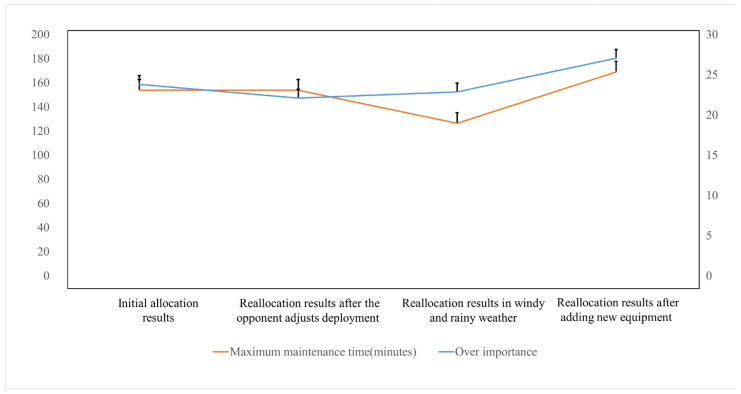
Line chart of changes in total importance and maximum maintenance time.

The changes in total equipment importance and maximum maintenance time are analyzed through Figure 5.

1. Adjustment of opponents deployment: When the threat threshold drops sharply, high-threat equipment such as E1 is abandoned. On the premise of ensuring the safety of maintenance units, core equipment is retained. Therefore, the time remains unchanged, but the importance decreases slightly.
2. Occurrence of windy and rainy weather: E7 is transferred from maintenance unit C to maintenance unit A, which not only avoids the fatigue coefficient of maintenance unit C exceeding the threshold but also shortens the task of maintenance unit C, reducing the maximum maintenance time from 153.92 to 126.51, and the equipment importance rises to 22.88, achieving the goal of reducing time and maintaining importance.
3. Addition of new damaged equipment: High-importance equipment such as E13 and E14 are prioritized for allocation within the constraints of spare parts and skills. Finally, the equipment importance increases by 18.6%, and the increase in maintenance time is controlled at 9.8%.

2. Analysis of maintenance time of maintenance units

**Figure 6:**
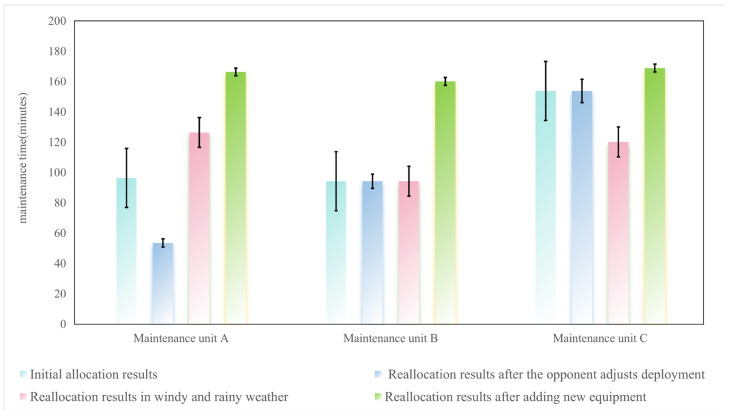
Bar chart comparing maintenance time of each maintenance unit.

The changes in maintenance time of each maintenance unit are analyzed through Figure 6.

1. Maintenance unit A: As an “elastic task unit”, it prioritizes receiving transferred tasks and new tasks with low threats and high matching degrees to ensure meeting the requirements of the overall scheme. In windy and rainy weather, it receives E7 transferred from maintenance unit C, increasing the task load and the maintenance time by 136%; when new damaged equipment is added, it receives E14, extending the task chain and increasing the maintenance time by another 31.6%.
2. Maintenance unit B: As a “core task unit”, it always retains high-value and low-volatility maintenance tasks. In the first two scenarios, the tasks received by maintenance unit B, such as E9, E6, and E2, are all high-value tasks, and the threat and fatigue constraints are satisfied, so the task chain remains unchanged; when new damaged equipment is added, it receives E13, increasing the task load and the maintenance time by 69.9%.
3. Maintenance unit C: As a “constraint-sensitive unit”, it actively reduces tasks when constraints are tightened and receives adaptive tasks when constraints are relaxed. When the opponent adjusts the deployment, maintenance unit C receives E8, E5, and E7, and the threat and fatigue satisfy the constraints, so the task chain remains unchanged; in windy and rainy weather, due to the fatigue threshold dropping to 0.2 caused by moderate rain, it abandons E7, reducing the task load and the maintenance time by 21.8%; when new damaged equipment is added, it receives E15, extending the task chain and increasing the maintenance time by 40.5%.

3. Analysis of equipment importance responsible for by maintenance units

**Figure 7:**
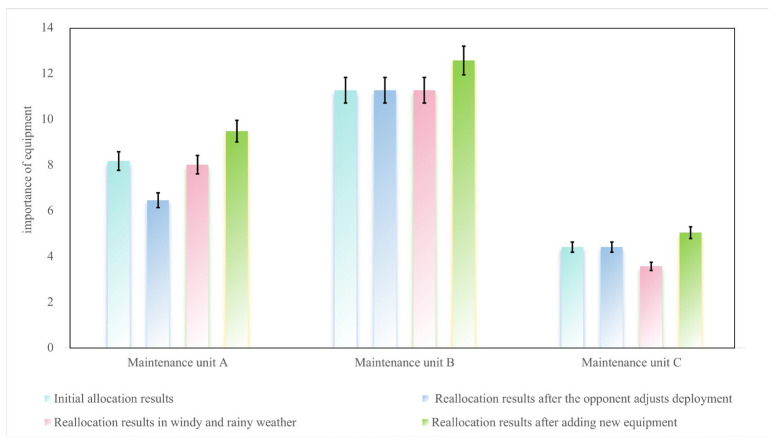
Bar chart comparing equipment importance responsible for by each maintenance unit.

The changes in equipment importance responsible for by each maintenance unit are analyzed through Figure 7.

1. Maintenance unit A: It prioritizes retaining and receiving high-importance equipment. When the opponent adjusts the deployment, it abandons E1, resulting in a 21% decrease in importance; when new damaged equipment is added, it receives E14, increasing the importance by another 18.4%.
2. Maintenance unit B: In the first two scenarios, it locks in task combinations with high synergy, such as E9 and E6. When new damaged equipment is added, it prioritizes allocating equipment with strong synergy with core tasks, such as E13 with a synergy importance of 0.4 with E6, increasing the equipment importance.
3. Maintenance unit C: When windy and rainy weather occurs, with tightened constraints, it prioritizes abandoning low-importance equipment such as E7, resulting in a 19.1% decrease in importance; in the scenario of adding new damaged equipment, it receives medium-value equipment adapted to remaining resources such as E15, increasing the importance by 41.3%.

The changes in each indicator reflect the synergistic effect of the dynamic scheduling strategy and the digital twin + robust optimization technology. The digital twin synchronizes the confrontation status in real time, providing data support for task adjustment; robust optimization quantifies uncertainty constraints, ensuring a balance between the applicability and importance of the adjustment scheme, and finally achieving efficient scheduling goals.

4. Analysis of Algorithm Performance Comparison

1. Convergence speed analysis: DT-DPSO has the fastest convergence because the digital twin synchronizes real-time data such as the status of wartorn equipment and maintenance resources, avoiding the algorithm from falling into invalid searches; moreover, the mechanism of combining individual optimal (pbest) and global optimal (gbest) in discrete particle swarm accelerates the approximation to the optimal solution. DPSO has the slowest convergence because it lacks real-time data support and needs to repeatedly iterate to verify the rationality of static data, resulting in reduced search efficiency. The convergence accuracy of DT-NSGAⅡ is lower than that of DT-DPSO because although the crossover mutation mechanism of the genetic algorithm can cover multiple solutions, it is less efficient than the directional search of particle swarm in meeting the demand for quickly focusing on the optimal solution in wartime scheduling.
2. Robustness analysis: DT-DPSO has the optimal robustness because the robustness optimization model quantifies the fluctuation ranges of four uncertain factors, and the digital twin corrects disturbance data in real time to ensure that the scheme still meets constraints under the worst-case scenarios. DPSO has the worst robustness because it does not consider the dynamic fluctuations of uncertain factors, and static schemes are prone to failure due to battlefield mutations. The feasibility rate of DT-NSGAⅡ is lower than that of DT-DPSO because although the multi-solution characteristic of the Pareto frontier can provide alternative schemes, it lacks the boundary constraints of robustness optimization when responding to uncertainties quickly, leading some schemes to exceed the capacity limit of maintenance units.
3. Dynamic reallocation speed analysis: DT-DPSO has the fastest dynamic response because during re-optimization, it reuses the individual optimal (pbest) and global optimal (gbest) records from the initial optimization, and the population size is reduced to 50, compressing the calculation amount to 60% of the initial amount; at the same time, the digital twin updates the data after mutation in real time, avoiding the time consumption of data preprocessing. DPSO and DT-NSGAⅡ have slower responses: DPSO needs to reinitialize the population to verify the mutated data, and DT-NSGAⅡ needs to recalculate the Pareto frontier, both of which increase the reallocation time.

## 9. Conclusions

This paper proposes a maintenance task scheduling method for battle-damaged equipment based on DT and robust optimization. Through the dynamic optimization mechanism of virtual–real interaction, it effectively solves the problems of poor adaptability of traditional scheduling methods to changes in uncertain factors and difficulty in dealing with emergency scenarios. Firstly, it realizes the real-time data synchronization between physical entities and virtual models through DT technology, solving the problem of information lag in traditional scheduling. Secondly, it quantifies four types of uncertain factors, such as combat intensity, equipment task matching coefficient, maintenance unit fatigue coefficient, and environmental threat, and constructs a robust optimization model to solve the problem of poor risk resistance of traditional models. Thirdly, it designs a dynamic reallocation mechanism and a discrete particle swarm optimization algorithm for solving, so as to solve the problem of insufficient dynamic adaptability of traditional schemes. Through simulation tests, the three indicators of the algorithm are compared. DT-DPSO performs the best in the maintenance scheduling of battle-damaged equipment: its convergence speed is 33.3% faster than that of DPSO and 20% faster than that of DT-NSGAII; its robustness is 16.3% higher than that of DPSO and 10.7% higher than that of DT-NSGAII; its dynamic reallocation speed is more than 40% faster than that of DPSO and more than 30% faster than that of DT-NSGAII. This method is suitable for maintenance scheduling requirements of high speed, stability, and anti-interference.

## Figures and Tables

**Table 5:** Initial allocation results of maintenance tasks.

Maintenance Unit	Equipment Number (in Maintenance Order)	Skill Matching	Spare Part Consumption (Types 1–4)	Maximum Fatigue Coefficient	Maximum Threat Coefficient	Maintenance Time (Minutes)	Equipment Importance
A	E4, E10, E1	Yes	[4, 5, 0, 3]	0.35	0.15, 0.2, 0.3	96.51	8.16
B	E9, E6, E2	Yes	[4, 6, 4, 0]	0.35	0.4, 0.2, 0.15	94.33	11.26
C	E8, E5, E7	Yes	[2, 7, 0, 3]	0.35	0.5, 0.5, 0.15	153.92	4.4
Unmaintained	E3, E11, E12						
Remarks	Maximum maintenance time: 153.92; total equipment importance: 23.82						

**Table 6:** Reallocation results after the opponent adjusts deployment.

Maintenance Unit	Equipment Number (in Maintenance Order)	Skill Matching	Spare Part Consumption (Types 1–4)	Maximum Fatigue Coefficient	Maximum Threat Coefficient	Maintenance Time (Minutes)	Equipment Importance
A	E4(√), E10	Yes	[4, 3, 0, 0]	0.2	0.2	53.6	6.45
B	E9(√), E6, E2	Yes	[4, 6, 4, 0]	0.35	0.2, 0.15	94.33	11.26
C	E8(√), E5, E7	Yes	[2, 7, 0, 3]	0.35	0.4, 0.15	153.92	4.4
Unmaintained	E1, E3, E11, E12						
Remarks	Maximum maintenance time: 153.92; total equipment importance: 22.11						

**Table 7:** Reallocation results in windy and rainy weather.

Maintenance Unit	Equipment Number (in Maintenance Order)	Skill Matching	Spare Part Consumption (Types 1–4)	Maximum Fatigue Coefficient	Maximum Threat Coefficient	Maintenance Time (Minutes)	Equipment Importance
A	E4(√), E10(√), E1, E7	Yes	[4, 5, 0, 3]	0.5	0.3, 0.15	126.51	8
B	E9(√), E6(√), E2	Yes	[4, 6, 4, 0]	0.35	0.15	94.33	11.26
C	E8(√), E5(√)	Yes	[2, 4, 0, 3]	0.2	0	120.29	3.56
Unmaintained	E3, E11, E12						
Remarks	Maximum maintenance time: 126.51; total equipment importance: 22.88						

**Table 8:** Reallocation results after adding new equipment.

Maintenance Unit.	Equipment Number (in Maintenance Order)	Skill Matching	Spare Part Consumption (Types 1–4)	Maximum Fatigue Coefficient	Maximum Threat Coefficient	Maintenance Time (Minutes)	Equipment Importance
A	E4(√), E10(√), E1(√), E7, E14	Yes	[4, 5, 0, 6]	0.5	0.15, 0.15	166.44	9.47
B	E9(√), E6(√), E2(√), E13	Yes	[4, 6, 7, 3]	0.5	0.3	160.25	12.56
C	E8(√), E5(√), E15	Yes	[3, 4, 3, 3]	0.35	0.2	169.02	5.03
Unmaintained	E3, E11, E12, E16						
Remarks	Maximum maintenance time: 169.02; total equipment importance: 27.06						

**Table 9:** Parameter settings of the three algorithms.

Algorithm Name	Population Size	Maximum Number of Iterations	Specific Parameter Settings	Termination Condition	Digital Twin Data Sampling Frequency
DT-DPSO	80	200	Inertia weight w: linearly decreasing from 0.4 to 0.9; learning factor $c_{1}=c_{2}=2$; disturbance trigger threshold: no update of gbest for five consecutive generations	200 iterations or gbest fitness fluctuation < 0.01	100 ms/time
DPSO	80	200	Inertia weight w: linearly decreasing from 0.4 to 0.9; learning factor $c_{1}=c_{2}=2$; no disturbance mechanism	200 iterations or gbest fitness fluctuation < 0.01	None (static initial data)
DT-NSGAⅡ	80	200	Crossover probability $P_{c}$= 0.9; mutation probability $P_{m}$ = 0.1; crowding threshold: 0.1	200 iterations or stable Pareto frontier	100 ms/time

**Table 10:** Convergence speed comparison of the three algorithms.

Algorithm Name	Number of Iterations to Reach Stable Fitness (Generations)	Convergence Accuracy	Initial Allocation Calculation Time (Seconds)
DT-DPSO	80	0.923	8.0
DPSO	120	0.857	9.2
DT-NSGAⅡ	100	0.889	10.0

**Table 11:** Robustness comparison of the three algorithms.

Algorithm Name	Standard Deviation of Total Equipment Importance	Standard Deviation of Maximum Maintenance Time (Minutes)	Scheme Feasibility Rate Under Uncertain Disturbances (%) *
DT-DPSO	0.14	2.29	96.5
DPSO	1.26	4.94	80.2
DT-NSGAⅡ	1.05	4.00	85.8
* Note: Uncertain disturbance scenarios include ±20% fluctuation in combat intensity, ±30% fluctuation in threat coefficient, and ±25% fluctuation in fatigue coefficient. Each scenario was tested 10 times, and the average feasibility rate was taken.			

**Table 12:** Dynamic reallocation speed comparison of the three algorithms.

Algorithm Name	Reallocation Time in Scenario of New Wartorn Equipment (Seconds)	Reallocation Time in Scenario of Enemy Deployment Adjustment (Seconds)	Reallocation Time in Windy and Rainy Weather Scenario (Seconds)
DT-DPSO	5.0	4.2	4.5
DPSO	8.3	7.8	7.5
DT-NSGAⅡ	7.1	6.5	6.8

## Data Availability

Data is unavailable due to privacy or ethical restrictions.
